# Embedding topography enables fracture guidance in soft solids

**DOI:** 10.1038/s41598-019-49986-1

**Published:** 2019-09-17

**Authors:** Christopher H. Maiorana, Mitchell Erbe, Travis Blank, Zachary Lipsky, Guy K. German

**Affiliations:** Department of Biomedical Engineering, Binghamton University, New York, 13902 USA

**Keywords:** Mechanical engineering, Soft materials

## Abstract

The natural topographical microchannels in human skin have recently been shown to be capable of guiding propagating cracks. In this article we examine the ability to guide fracture by incorporating similar topographical features into both single, and dual layer elastomer membranes that exhibit uniform thickness. In single layer membranes, crack guidance is achieved by minimizing the nadir thickness of incorporated v-shaped channels, maximizing the release of localized strain energy. In dual layer membranes, crack guidance along embedded channels is achieved via interfacial delamination, which requires less energy to create a new surface than molecular debonding. In both membrane types, guided crack growth is only temporary. However, utilizing multiple embedded channels, non-contiguous crack control can be maintained at angles up to 45° from the mode I fracture condition. The ability to control and deflect fracture holds great potential for improving the robustness and lifespan of flexible electronics and stretchable sensors.

## Introduction

Crack nucleation and dynamic fracture processes govern failure in a multitude of hard^1,2^ and soft materials^3,4^, ranging from biological tissues to polymeric elastomers. While studies have explored fracture propagation in both brittle^5^ and ductile^6–8^ materials, the means of controlling crack propagation remains relatively unexplored, despite the existence of numerous biological materials such as nacre^9,10^, human stratum corneum^3^, and dentin^11^, which exhibit microstructures that can guide fracture pathways. Crack guidance can impart beneficial mechanical qualities to materials, most notably increases in total crack path length that act to toughen the material^12^, and increase fatigue strength^13^. As such, biomimetic and bioinspired materials^14–16^ hold great potential for improving the robustness of structured materials^17^, composite materials^18–20^, manufacturing^21^, and flexible electronics^22,23^.

Prior studies have primarily explored the control of crack propagation in brittle materials^24^ such as silicon wafers^15,25^ and glass^12,14^, via the inclusion of defects^26,27^. Here, cracks propagate from defect to defect to alleviate high magnitude strain energies in their vicinity^28,29^. Yet, despite the recent attention that soft materials and thin compliant films have received due to their relevance to flexible electronics^22,23,30^, biomedical devices^21,23,31^, and both biofilm^32,33^ and tissue^34,35^ mechanical characterization, neither the failure mechanics of soft hyperelastics, nor the control of crack propagation has been studied in detail. In this study, we report on the use of incorporating and embedding topographical channels, similar to those present on the surface of human skin, to guide fracture in both single layer silicone elastomer membranes, and uniform thickness dual layer membranes that appear homogeneous.

## Topographical Microchannels

**Figure 1:**
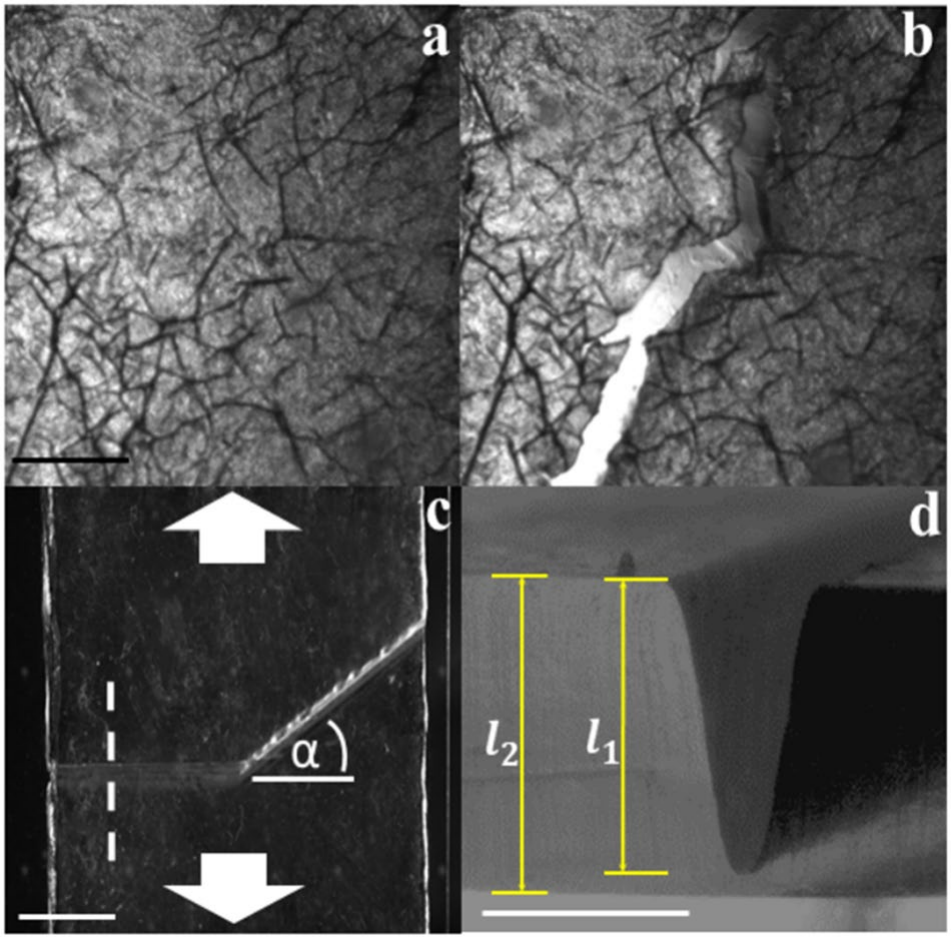
Topographical features in isolated human stratum corneum and silicone elastomer membranes. (**a**) Brightfield image of isolated human stratum corneum. The darker regions indicate natural topographical microchannels^3,52,53^. The scale bar denotes 500 µm. (**b)** Stratum corneum sample under a biaxial stress showing cracks propagating along the microchannels. (**c**) Plan view of a single layer elastomer membrane containing a topographical channel with a reorientation angle of *α* = 30° located at the midpoint of the membrane width. Arrows indicate the orientation of uniaxial loading. The dashed line indicates the location of the cross sectional profile shown in panel d. Scale bar denotes 5mm. (**d**) Cross sectional profile of a single layer elastomer membrane revealing the v-shaped channel. *l*_1_ and *l*_2_ respectively denote the channel depth and membrane thickness. The scale bar denotes 1 mm.

The outermost layer of human skin, or stratum corneum, exhibits a network of v-shaped topographical microchannels^3^, as highlighted by the brightfield image in Fig. 1a. When isolated samples of this tissue are biaxially strained, Fig. 1a,b reveal that propagating cracks continually reorient, closely following the topographical features. The microchannels therefore appear to be capable of guiding fracture^3^. We first study the ability to control fracture guidance by replicating topographical channels in silicone elastomer membranes. The membranes are created by curing the elastomer on 3D printed masks exhibiting v-shaped reliefs. Figure 1c shows a brightfield image of a membrane with the incorporated topographical channel. The channel, of cross sectional width ~0.8 mm, is comprised of two sections: one aligned parallel (denoted henceforth as the parallel section) with the membrane width, *w*, and an angled section oriented *α* = 30° to this direction (denoted henceforth as the angled section). This reorientation occurs midway across the width of the membranes. Figure 1d shows a brightfield image of the channel cross section. The channel extends a distance *l*_1_ into the membrane, of thickness *l*_2_.

**Figure 2:**
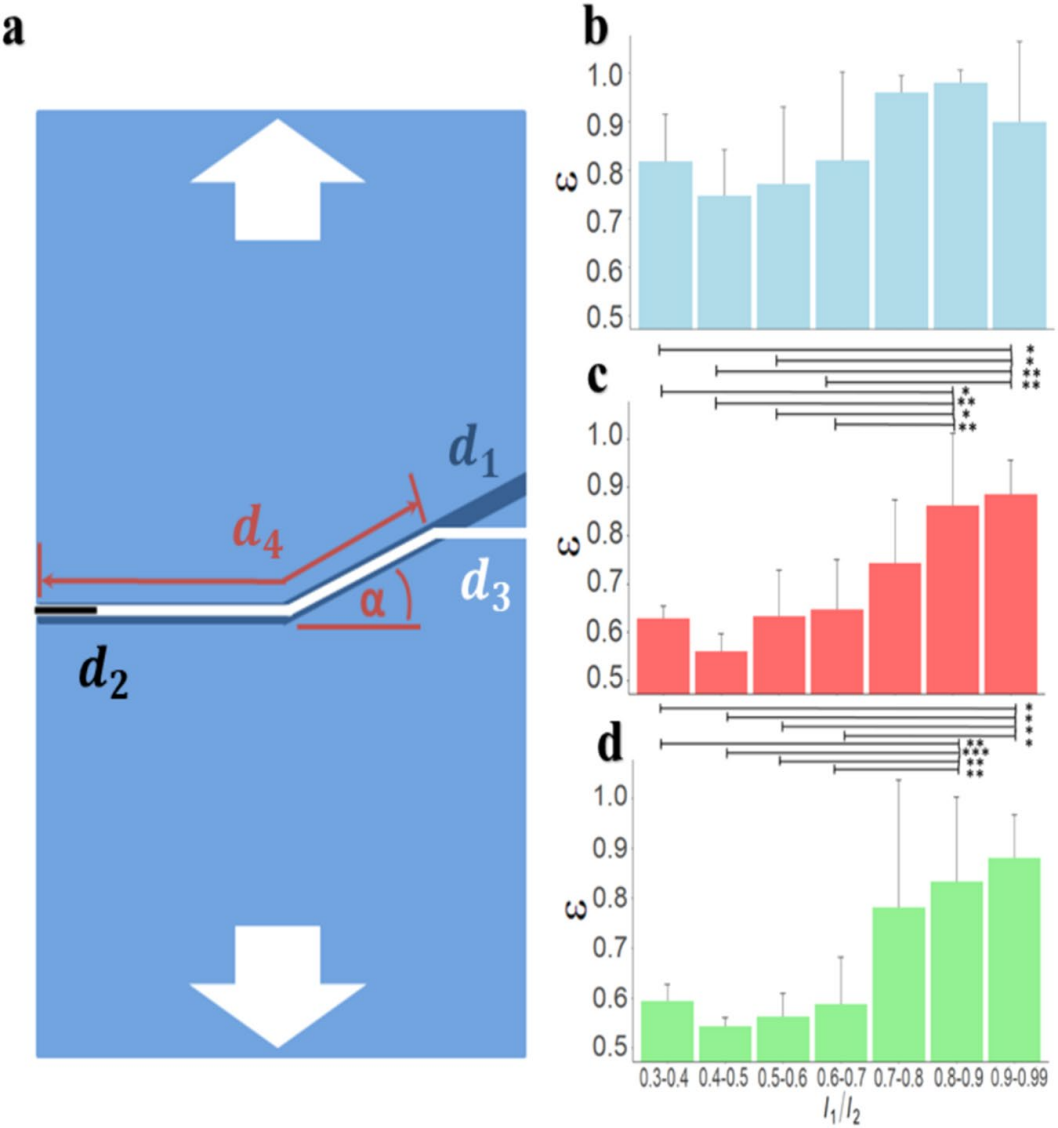
Crack propagation control in uniaxially loaded single layer membranes. (**a**) Schematic of crack propagation in single layer membranes containing a channel. Lines *d*_1_, *d*_2_, *d*_3_, and *d*_4_ respectively denote the total length of the topographical channel including parallel and angled sections, the initial notch length, the total crack path length, and the length of the crack path coincident with the topographical channel. The arrows indicate the orientation of uniaxially loading. (**b**–**d**) Average normalized fraction of crack pathway coincident with the channel, *ε* = *d*_4_/*d*_1_, plotted against normalized channel depth, *l*_1/_*l*_2_, for reorientation angles of: (**b**) *α* = 10°, where error bars denote the standard deviation of *n* = 3 individual samples for *l*_1_/*l*_2_ = *0*.*4–0*.*5*, *0*.*7–0*.*8*, *0*.*8–0*.*9*, *0*.*9–0*.*99*, *n* = 4 samples for *l*_1_/*l*_2_ = *0*.*3–0*.*4*, *n* = 5 samples for *l*_1_/*l*_2_ = 0.6–0.7, and *n* = 10 samples for *l*_1_/*l*_2_ = *0*.*5–0*.6 (**c**) *α* = 20°, where error bars denote the standard deviation of *n* = 3 individual samples for *l*_1_/*l*_2_ = *0*.*3–0*.*4*, *0*.*4–0*.*5*, *n* = 4 samples for *l*_1_/*l*_2_ = *0*.*5–0*.*6*, *0*.*9–0*.*99*, *n* = 6 samples for *l*_1_/*l*_2_ = *0*.*8–0*.*9*, *n* = 11 samples for *l*_1_/*l*_2_ = *0*.*7–0*.*8*, and *n* = 14 samples for *l*_1_/*l*_2_ = 0.6–0.7 (**d**) *α* = 30°, where error bars denote the standard deviation of *n* = 3 individual samples for *l*_1_/*l*_2_ =  *0*.*3–0*.*4*, *0*.*4–0*.*5*, *n* = 4 samples for *l*_1_/*l*_2_ = *0*.*7–0*.*8*, *0*.*9–0*.*99*, *n* = 10 samples for *l*_1_/*l*_2_ = *0*.*5–0*.6, *n* = 13 samples for *l*_1_/*l*_2_ = *0*.*6–0*.*7*, and *n* = 14 samples for *l*_1_/*l*_2_ = *0*.*8–0*.*9*. P-values in (**b**) are established using a 1-way ANOVA with Welch’s F correction with an F-value of 1.8, (**c**)through a 1-way ANOVA obtaining an F-value = 6.1 and **d** using a 1-way ANOVA with Welch’s F correction and an F-value of 8.7.

Elastomer membranes with varying dimensionless channel depths, *l*_1/_*l*_2_, channel reorientation angles, *α*, and overall channel lengths, *d*_1_, are fabricated. In each case, a notch of length *d*_2_ = 2 mm is made using a razor blade at the edge of the parallel section, as shown in Fig. 2a. Lengths *d*_1_, *d*_2_, *d*_3_, and *d*_4_ respectively denote: the total length of the topographical channel including parallel and angled sections, the initial notch length, the total crack path length, and the length of the crack path coincident with the topographical channel. Membranes are then uniaxially strained at a rate of $\dot{\gamma }=0.029$ s^−1^ until complete rupture occurs. The impact of the channel reorientation and dimensionless channel depth on crack propagation is first assessed by quantifying the ratio of the crack path coincident with the channel, to the overall channel length, $\varepsilon ={d}_{4}/{d}_{1}$. Figure. 2b–d respectively show changes in $\varepsilon$ with *l*_1/_*l*_2_ for reorientation angles of *α* = 10°, 20° and 30°. Dimensionless channel depths of *l*_1/_*l*_2_ < 0.7 result in cracks that predominantly ignore the reorientation of the channel. Here, cracks propagate primarily across the membrane width, and remain perpendicular to the applied uniaxial stress, consistent with mode I cracking^36,37^ (the “opening” mode, where the crack plane is normal to the applied force). However, with *l*_1/_*l*_2_ < 0.8, cracks readily propagate along the reoriented channel. As the crack changes direction, the mode of fracture transitions from preferential mode I failure to mixed mode I and II (mode II occurring when the applied force is parallel to the crack plane, mixed mode I and II occurring when the applied force acts at a complex angle between 0° and 90° from the crack plane)^36,38,39^, which we denote as guided fracture. Within this regime, monotonic increases in $\varepsilon$ with *l*_1/_*l*_2_ occur for the larger reorientation angles of *α* = 20° and 30°. The maximum extent to which cracks follow the channel also decreases as $\alpha$ is increased. We note that for small reorientation angles of *α* = 10°, values of $\varepsilon$ remain relatively large, regardless of the topographical depth.

Within the regime $0.5 < \varepsilon  < 1,$crack reorientation does occur, however cracks do not propagate fully along the angled channel. Once guided fracture is lost, cracks moves laterally away from the channel, realigning perpendicularly to the applied uniaxial stress. This agrees with previous studies of mixed mode I and II failure, where reoriented cracks asymptote towards an alignment perpendicular to the maximum tensile stress^36,38,39^. With angled sections all reoriented less than 45° to the applied stress, the uniaxial stress at the crack tip will remain greater than the shear components^40^.

**Figure 3:**
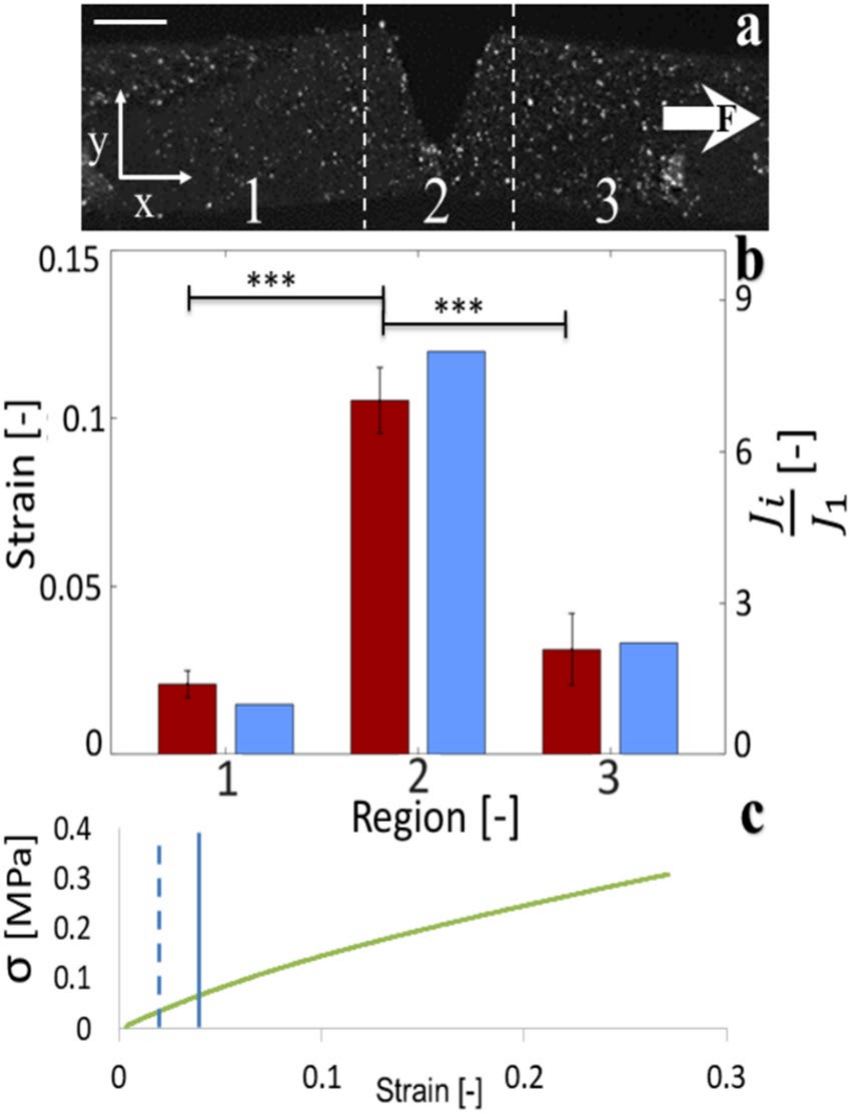
Heterogeneous strains in the vicinity of topographical channels. (**a**) Reflectance image of a graphite coated membrane under a uniaxial stress of *α* = 30 kPa. The scale bar denotes 1 mm. Region 2 shows the location of the topographical v-shaped channel. Graphite particles, visible as white speckles on the membrane surface, are used to track spatially resolved displacements. (**b**) Average measured strains (red or darker bars) for the three regions along the membrane. Error bars indicate standard deviations of n = 3 different particle pair strain measurements, taken from within each of the three regions. P-values were established using a 1-way ANOVA obtaining an F-value of 40.9. The corresponding approximate elastic potential strain energy ratio (blue or light gray bars) is displayed for each of the three regions. Strain energies in each region are scaled by the strain energy of region 1, located closest to the stationary tensometer grip. (**c**) Engineering stress vs strain plot for a 5:1 base to curer ratio PDMS elastomer sample of size 19 × 49 × 2 mm containing no topographical inclusions. The solid vertical line indicates the proportional limit. The dashed vertical line denotes the applied global average strain in the membrane when spatially resolved strains are established.

Crack guidance in the membrane channels can be explained through release of elastic strain energy, the driving force behind cracking^41–44^. Initially, cracks propagate along the base of the parallel v-shaped channel, perpendicular to the applied tensile stress^45^. The reflectance image in Fig. 3a shows a profile view of a membrane coated with graphite powder under a uniaxial stress of σ = 30 kPa and an average macroscopic tensile engineering strain of $\gamma =0.02$. The central region contains the topographical channel. Figure 3b compares measured local strains on either side of the channel (regions 1 and 3), and at the lowest point along the channel, referred to as the channel nadir (region 2). The largest strains occur at the nadir of the channel. Here, all strains except the strain at the channel nadir fall below the proportional limit (Fig. 2b). Approximating the local strain energy density in each of the regions *i* = 1−3 as $\,{W}_{i}=\frac{1}{2}E{\gamma }^{2}$, where the elastic modulus of the PDMS is *E* = 1.48 MPa, we establish the elastic energy stored locally in each region, scaled by that stored in region 1 using the expression, *J*_*i*_/*J*_1_ = *W*_*i*_/*t*_*i*_ = *W*_1_/*t*_1_, where the local thickness of the membrane, *t*_*i*_, is *t*_1_ = *t*_3_ = *t*_2_ and *t*_2_ = *t*_2_ − *t*_1_. Here, *J*_2_/*J*_1_ = 7.9, and *J*_3_/*J*_1_ = 2.2, indicating that the greatest release of strain energy, where rupture would occur^43^, would confine cracking within the channel. We anticipate cracks remain confined in the reoriented channels until the strain energy release in region 1 or 3 surpasses that in region 2.

## Dual Layer Membranes

Motivated by the need to print flexible electronics on flat, rather than topographically heterogeneous substrates, we further consider the ability to direct fracture in uniform thickness membranes in which topographical features are embedded. Here dual layer membranes are created, such that the membrane exhibits apparent homogeneity and uniform thickness. This secondary layer allows for greater versatility as a printable surface, as indeed, printing a conformant film on a topographically featured surface such as human skin limits the layered film to thicknesses less than 5 µm^46^.

**Figure 4:**
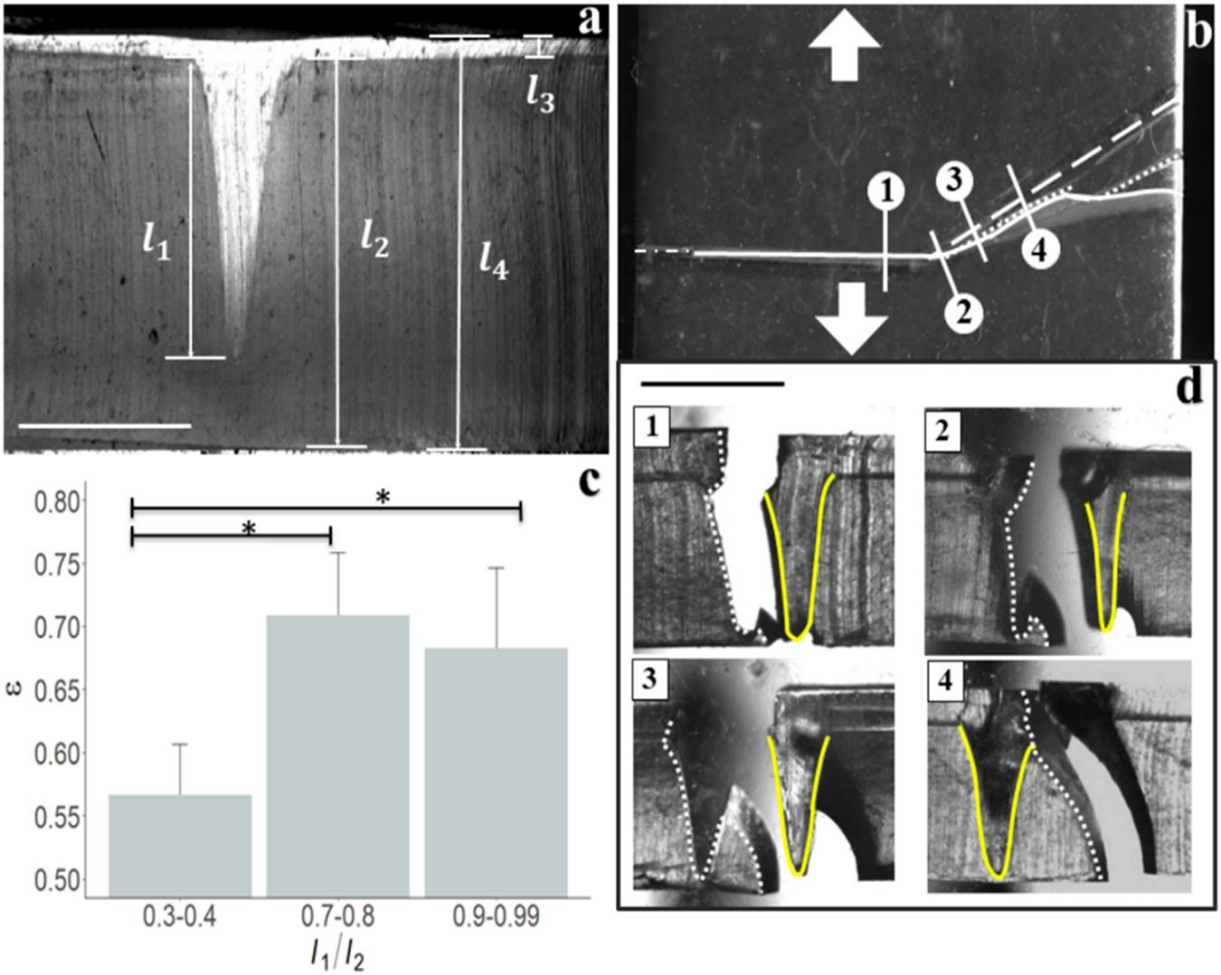
Crack propagation and fractography in uniform thickness dual layer membranes. (**a**) Cross sectional profile of a uniform thickness dual layer elastomer membrane exhibiting an embedded channel. Here, *l*_2_ and *l*_1_ respectively denote the overall primary layer thickness and depth of channel into this layer. *l*_3_ and *l*_4_ denote the secondary layer thickness and overall membrane thickness. The scale bar denotes 1 mm. (**b**) Plan view of a dual layer membrane overlaid with lines depicting the location of the embedded angled channel (dashed line), the crack path at the base of the membrane (solid line), the initial 2mm notch (dash-dot white line), and the crack path at the top of the membrane (dotted line). Lines numbered 1 through 4 denote the locations of cross sectional images shown in panel d. Arrows indicate the direction of applied stress. The black scale bar denotes 5 mm in panel b. (**c**) Average normalized fraction of crack pathway coincident with the channel, *ε*, for *α* = 30°, plotted against normalized channel depth *l*_1/_*l*_2_ Error bars denote standard deviations of *n* = 4 substrates. P-values are calculated using a 1-way ANOVA with an F-value of 8.3 (**d**). Brightfield images of cross sectional images labelled in panel b, showing lateral movement of delamination (dotted white lines) around the embedded channel (solid yellow line).

Figure 4a shows a cross sectional schematic of a dual layer membrane, formed through additive manufacturing. The base layer, of thickness, *l*_2_, and channel depth, *l*_1_, is fabricated in the same manner as the single layer membranes. However, spin coating and curing a second elastomer layer with thickness, *l*_3_, and fracture strain the same as the base layer, embeds the topographical channel within the uniform thickness (*l*_4_) membrane. To better compare crack guidance with single layer membranes we maintain the use of *l*_1/_*l*_2_ rather than *l*_1/_*l*_4_ to characterize channel depth, given that ${l}_{2}\approx {l}_{4}$.

A representative schematic of failure in these membranes is provided in Fig. 4b. Cracks propagate initially under mode I failure, perpendicular to the applied stress. At the site of the channel reorientation, cracks reorient, follow the angled channel for a limited distance, then move laterally away from the embedded channel, similar to the observed behavior in single layer channels. The ability of these membranes to guide crack propagation is evaluated in Fig. 4c. Crack guidance does occur, with statistically significant increases in *ε* occurring when the dimensionless channel depth reaches *l*_1/_*l*_2_ > 0.7 for reorientation angles of 30°. However, further increases in *l*_1/_*l*_2_ does not further improve the ability to guide cracking. In order to better understand how crack guidance is achieved here, cross sectional images of the membranes at the specified locations in Fig. 4b are obtained and shown in Fig. 4d. These images confirm that cracks propagate via delamination of the two layers at the location of the embedded channel. Initially, cracks propagating along the parallel channel occur from delamination at the channel wall side facing the moving tensometer grip (top of Fig. 4b). Once the crack reaches the angled segment, cracks reorient, and the transition to mixed mode failure causes the delamination site to progress around the edge of the channel, from the wall facing the moving tensometer grip to the wall facing the stationary grip (bottom of Fig. 4b). This is analogous to the lateral deviation expected in cracks propagating under mixed mode fracture. Once the site of delamination fully progresses around the channel wall, cracks eventually move laterally away from the channel, resulting in a loss of crack guidance. Cracks then reorient and asymptotically realign perpendicular to the applied uniaxial stress.

The cause of crack guidance in these membranes initially appears to be a reduced energy cost of creating a new surface at the interface between the two layers. To verify this, uniaxial tensometry is used to compare the fracture toughness of a homogeneous elastomer film to a membrane of equivalent dimensions containing a vertical interface across the membrane width. This is achieved by placing a uniform thickness elastomer membrane in a rectangular mould, removing half with a razor blade, refilling the volume with uncured elastomer, and finally curing the membrane. Cracking in the membrane that contains an interface occurs entirely along the interfacial plane, with a work of fracture per unit crack length of *W*_*f*_ = 3 Jm^−1^. In comparison, rupture in the homogenous membrane requires *W*_*f*_ = 20 Jm^−1^ for a near identical total crack length. This confirms that the interface provides an energetically preferential pathway for cracks to propagate^4^, over the more costly molecular debonding of the bulk material.

## Additional Factors Controlling Fracture

**Figure 5:**
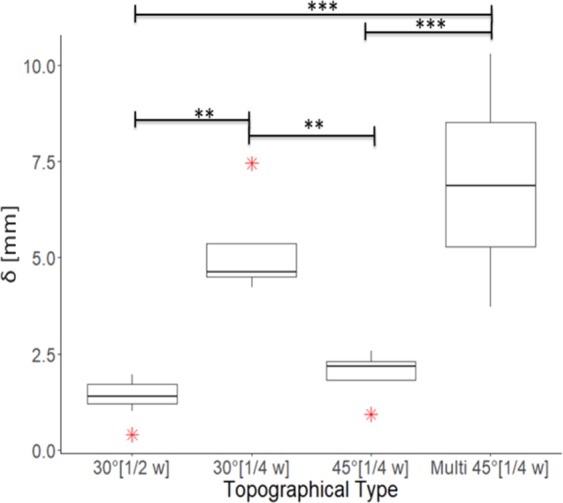
Guided crack deviation in dual layer membranes. Box plot showing the longitudinal distance along the length of the uniaxially strained membrane that cracks reach during rupture, *δ*, plotted against the type of embedded topography. Topographical types are distinguished on the horizontal axis by the reorientation angle in degrees, followed by the lateral position across the membrane width, *w*, where the reorientation occurs (in square brackets). The topographical type labelled with Multi denotes membranes containing 9 parallel angled sections separated by 1mm. Dark horizontal lines within each box denotes the median of *n* = 4 individual membranes. Whiskers extend to the minimum and maximum of each data set. Box heights denote the inter-quartile range. Red star symbols denote outliers. P-values are calculated using a 1-way ANOVA with Welch correction obtaining an F-value of 10.9.

In order to establish if additional factors impact the ability to guide cracks, the influence of crack velocity at the site of channel reorientation is next assessed. Supplemental Fig. S1 highlights that as cracks propagate along the parallel channel section of a dual layer membrane, the tip velocity notably increases. We anticipate that this increased momentum may prevent cracks from reorienting. Indeed, evidence for this exists, with previous studies reporting that higher velocity cracks propagating in both bone and soft gels extend irrespective of material structure, and display smoother crack interfaces^47,48^. Smoother crack interfaces are also observed at higher velocities in the dual layer membranes. We control the crack velocity at the location of the channel reorientation site by varying the lateral position of the reorientation. Here, crack guidance is quantified by measuring the average longitudinal distance along the dual layer membrane length between the initial and final crack tip positions, *δ*. Figure 5 first shows that by repositioning the reorientation angle of 𝛼 = 30° from midway across the membrane width (1/2 w) to a quarter of the membrane width (1/4 w) results in more than a two-fold increase in δ, indicating that the reorientation of propagating cracks is more easily achieved at lower crack velocities.

**Figure 6:**
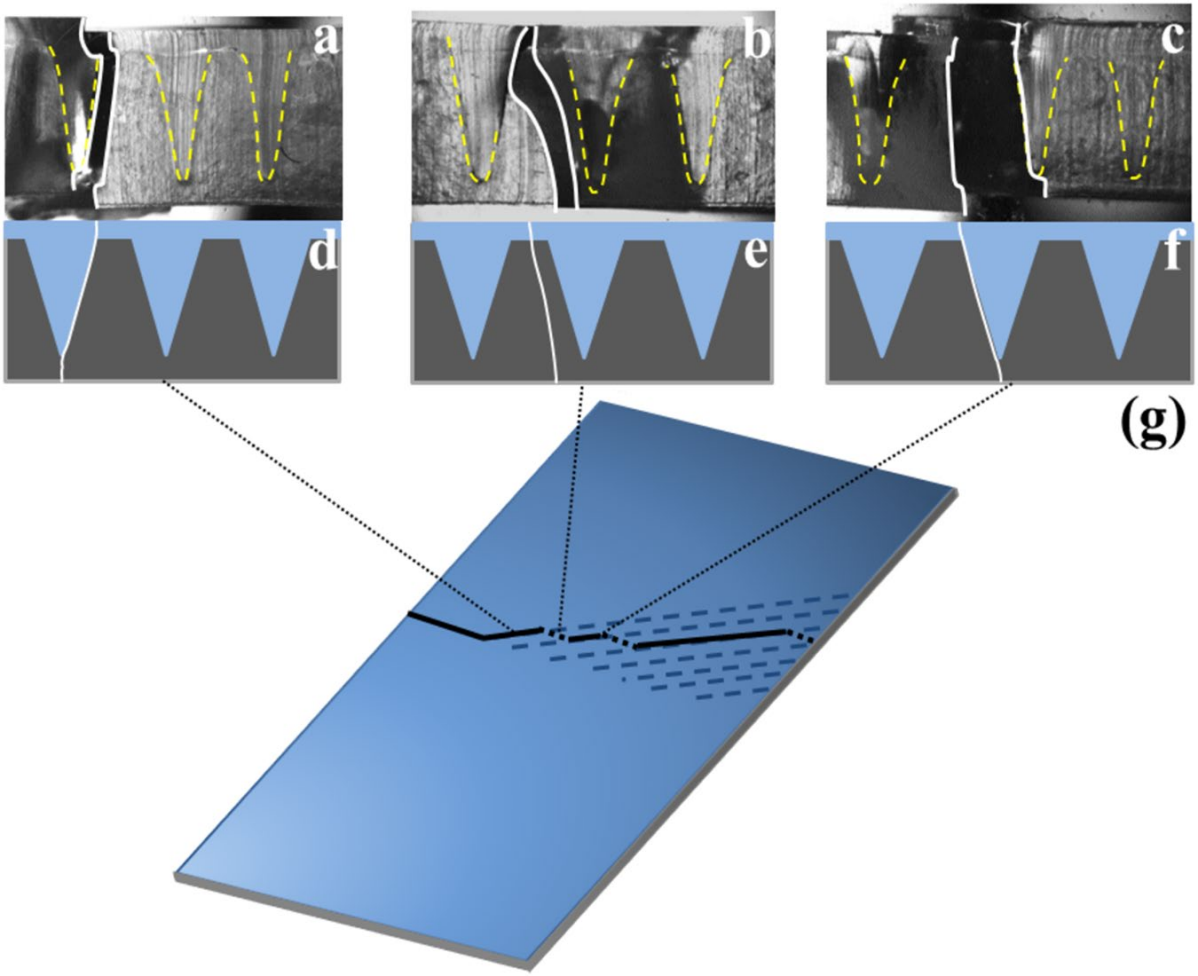
Schematic and fractography of a dual layer membrane containing multiple angled sections in parallel. (**a**–**c**) Cross-sectional brightfield images of a fractured dual layer membrane at the positions located on the schematic in panel g. Yellow dotted lines outline the embedded topographical channels. White solid lines indicate the location of the delamination and fracture surfaces. (**d**–**f**) Schematic representations of fracture behavior shown in panels (**a**–**c**). Solid white lines denote the representative location of the delamination/fracture surface. (**g**) Schematic of the dual layer membrane. Dashed blue lines denote the locations of embedded channel angled sections. Solid black lines indicate representative regions where crack guidance occurs. Dotted lines indicate regions of uncontrolled fracture.

With the reorientation position maintained at a quarter of the membrane width, an increase in the reorientation angle to 𝛼 = 40° results in a notable decrease in *δ*. This decreased fracture guidance with increasing reorientation angle is consistent with the results of single layer membranes (Fig. 2). Remarkably however, when multiple aligned angled channels are embedded in the dual layer membrane with the reorientation position fixed at a quarter width, crack deviations, *δ*, dramatically increase relative to the 30° reorientation angle. Cross sectional images shown in Fig. 6a–c highlight that the additional angled channels embedded in the membrane act as fail-safes. Crack guidance is achieved through the same process as the dual layer membranes with single embedded channels. However here, once crack guidance in an angled channel is lost due to lateral propagation of the delamination around the channel width, the crack propagates laterally away from the channel and encounters another, along which crack guidance is re-established. A schematic representation of this process is provided in Fig. 6d–f. This non-contiguous crack guidance enables cracks to deviate laterally by up to half the channel width until complete membrane rupture. However, we anticipate through closer spacing of the angled channels, guidance might be improved. Supplemental Movie 1 shows crack guidance within membranes containing multiple angled channels. Interestingly, the topographical inclusions do not degrade the mechanical toughness of the membranes. Supplemental Fig. S2 highlights that the fracture toughness of membranes with topographical inclusions (7.5 ± 7.2 Jm^−3^, *n* = 4 membranes) does not statistically differ from that of homogenous membranes without embedded channels (3.1 ± 0.95 Jm^−3^, *n* = 4 membranes). We attribute this to increases in the overall crack path length raising the overall energy cost of creating the new surfaces^9^.

## Concluding Remarks

In this article, we demonstrate that crack guidance can be achieved in compliant and hyperelastic materials. In single layer membranes, the inclusion of topographical channels with depths upwards of 70% of the total membrane thickness can cause cracks to reorient at oblique angle of up to 30°. In dual layer membranes with uniform thickness and apparent homogeneity, embedded channels cause cracks to propagate along the interface between the two layers. The primary difference between the two types of membranes is that while single layer membranes exhibit non-uniform thickness and crack propagation along the channel nadir prior to reorientation, dual layer membranes exhibit uniform thickness, with fracture occurring through delamination of the two layers. Embedding multiple channels enables non-contiguous crack control at oblique angle of up to 45°. Moreover, the inclusion of embedded topographical features does not reduce the mechanical toughness of the membranes.

The ability to control and deflect fracture holds great potential for the flexible electronics and wearable sensors community, where avoiding fracture in regions crucial to device function is critical to improving the durability and lifespan of compliant platforms that undergo large deformations. With current 3D printing and nanofabrication techniques able to create morphologies with increasing spatial resolutions^49,50^, future work should explore if crack guidance can be achieved in significantly thinner membranes, and explore if patterned networks of embedded microchannels within the uniform thickness membranes can be used to further increase the toughness of materials to values greater than their homogeneous equivalents.

## Methods

### Fabrication of single layer flexible substrates

A substrate comprised of a flat base and v-shaped relief is first 3D printed (Objet30 Pro, Stratasys, Eden Prairie, MN), then coated with 1% weight per volume of polyvinyl alcohol (PVA, Sigma-Aldrich, St Louis, MO) in deionized water. After drying for 2 hr at 60 °C, the solution forms a thin homogeneous solid coating. The substrate is then placed with the raised side facing upwards in a custom mould with an aluminium base and acrylic walls. Intersections of the base and walls are then sealed with vacuum grease. A silicone elastomer is prepared by mixing a base (Sylgard 184, Dow Corning, Midland, MI) with the curing agent in weight ratio of 5:1. After mixing and degassing, the elastomer is poured into the chamber. A flat ended aluminium press is then used to squeeze the elastomer to the desired thickness. During this process, excess elastomer escapes from the chamber through two ports located at the ends of the aluminium press. After fully curing the elastomer at 40 °C for 12 hr, the chamber is submerged in deionized water. A metal spatula is then used to delaminate the elastomer from the mould. Here, the PVA coating reduces adhesion forces between the cured elastomer and substrate. The cured elastomer membrane exhibits a v-shaped channel, of cross sectional width ~0.8 mm, with two contiguous sections; a channel perpendicular to the long axis of the elastomer (denoted as the parallel section), and a channel at an oblique angle to this orientation (denoted as the angled section). Bright-field microscopy (Nikon Eclipse Ti-U, Nikon, Melville) using a 1X objective lens with 0.4 numerical aperture is then used to measure the thickness and channel depth of each membrane. Samples are cut to identical dimensions of 19 × 49 mm (width × length). Membrane thicknesses vary between 1.8 and 2.2 mm.

### Fabrication of uniform thickness dual layer membranes

The base layer of the membrane is first created using the previously described method for single layer elastomers. However, 3% fumed silica by mass (Sigma-Aldrich, St Louis, MO) is mixed into the uncured elastomer so that the different membrane layers can be visibly distinguished. While the inclusion of the silica does increase the Young’s modulus of the elastomer layer, it does not alter the strain at which fracture occurs, as shown in Supplemental Fig. S3. A 5:1 ratio of silicone elastomer base to curer is then mixed, degassed and spin coated onto the base layer at 300 rpm for 20 sec. This second layer readily fills the topographical channels in the base layer. The composite membrane is then cured at 110 °C for 10 min. The uniformity of the overall membrane thickness and channel depth is measured using bright-field microscopy (Nikon Eclipse Ti-U, Nikon, Melville). Variations in thickness do not exceed 7%.

### Fabrication of homogeneous membranes and those with vertical interfaces

Homogenous silicone elastomer membranes are prepared using the previously described method without the 3D printed substrate inserted into the mould. The same process is used to make membranes with vertical interfaces. However, once the homogenous membrane is fully cured, an incision is made midway along the length of the substrate using a razor blade. Half of the cured elastomer is then removed. The empty region is then refilled with elastomer to the height of the cured section and allowed to cure. The resulting membrane is then removed from the casing and cut to a dimension of 19 × 49 mm.

### Fracture experiments

Individual membranes are clamped in a tensometer (Instron 3342 with 500 N load cell, Norwood, MA), with an initial grip separation of 35 mm. Samples are strained at a rate of 1mms^−1^ until complete membrane rupture occurs, equivalent to a strain rate of $\dot{\gamma }=0.029$ s^−1^. Prior to straining, a 2 mm notch is made at the edge of the parallel channel section in both the single and dual layer membranes. Force versus displacement data is obtained at a frequency of 10 Hz as membranes are strained up to complete rupture. Membrane dimensions are used to convert this data to engineering stress and strain. An algorithm written in MATLAB is used to extract crack tip velocities during membrane rupture, captured (iPhone SE, Apple Cupertino, CA) at 240 frames per second at a minimum resolution of 50 μm/pixel.

### Cross sectional imaging

Membranes are sectioned after rupture using a paper trimmer (Swingline, Lincolnshire, IL). Cut planes of each section are oriented perpendicular to the long axis of the incorporated or embedded topographical channel. Individual sections are placed on a glass coverslip, imaged in bright field using an inverted microscope (Nikon Eclipse Ti-U, Nikon, Melville) with 1X objective lens and captured using a CCD camera (Andor Clara. Belfast, Northern Ireland).

### Sample imaging during strain testing

During mechanical testing, membranes with a single parallel channel extending fully across the membrane, are imaged at a frequency of 500 Hz using a high speed camera (Cooke Pro, pco.1200 s, Germany) with 35 mm extension tube and zoom lens (Navitar Zoom 7000). Local v-shaped channel deformations are captured at a resolution of 29.32 μm/pixel (1280 × 1042 pixels). To enable tracking of localized strains, membranes are coated with graphite powder. Membranes used for this study have an elastic modulus of *E* = 1.48 MPa (Supplemental Fig. S1) formed using a 5:1 base to curer ratio of Sylgard 184 containing 3% fumed silica by mass.

### Strain quantification

Spatially resolved 2D displacements surrounding the incorporated parallel topographical channel are quantified with single pixel resolution using a centroid tracking algorithm^51^. The longitudinal positions of particle centroids along the long axis of the membrane are used to establish displacements. Local tensile strains are established from particle pairs using the expression, ${\gamma }_{x,ij}=(\mathop{{u}_{i}}\limits^{\rightharpoonup }-\mathop{{u}_{j}}\limits^{\rightharpoonup })/(\mathop{{x}_{i}}\limits^{\rightharpoonup }-\mathop{{x}_{j}}\limits^{\rightharpoonup })$, where $\mathop{u}\limits^{\rightharpoonup }$ and $\mathop{x}\limits^{\rightharpoonup }$ respectively denote the longitudinal displacement and position vectors of particles i & j. To prevent anomalous strains arising from the tracking resolution, only the longitudinal strains quantified from particle pairs with an initial proximity greater than 10 pixels(300 μm) are quantified. Individual pair strains are calculated for regions aligned with the channel nadir, and either side of the channel, a minimum of 1 mm away from the channel edge.

### Statistical analysis

Statistical analyses are performed using R Version 3.4.2. To assess for normality and homoscedasticity, Shapiro-Wilke’s and Levene’s test are respectively performed for all data sets. Statistical significance is found using a one way analysis of variance (1-way ANOVA. Posthoc analyses are performed when statistical significance is established. In the figures, * denotes p ≤ 0.05, ** denotes p ≤ 0.01, and *** denotes p ≤ 0.001.

## Supplementary information

Supplemental Material

Supplemental Movie 1
