# Supplementary material for: Embedding topography enables fracture guidance in soft solids
Source: Sci Rep. 2019 Sep 17;9:13493. doi: 10.1038/s41598-019-49986-1 (PMC6748918; doi:10.1038/s41598-019-49986-1)
Supplement: Supplementary file 1 — Supplemental Material [file 41598_2019_49986_MOESM1_ESM.docx]

# Embedding topography enables fracture guidance in soft solids.

Christopher H. Maiorana, Mitchell Erbe, Travis Blank, Zachary Lipsky, and Guy K. German

# Supplemental figures and movies


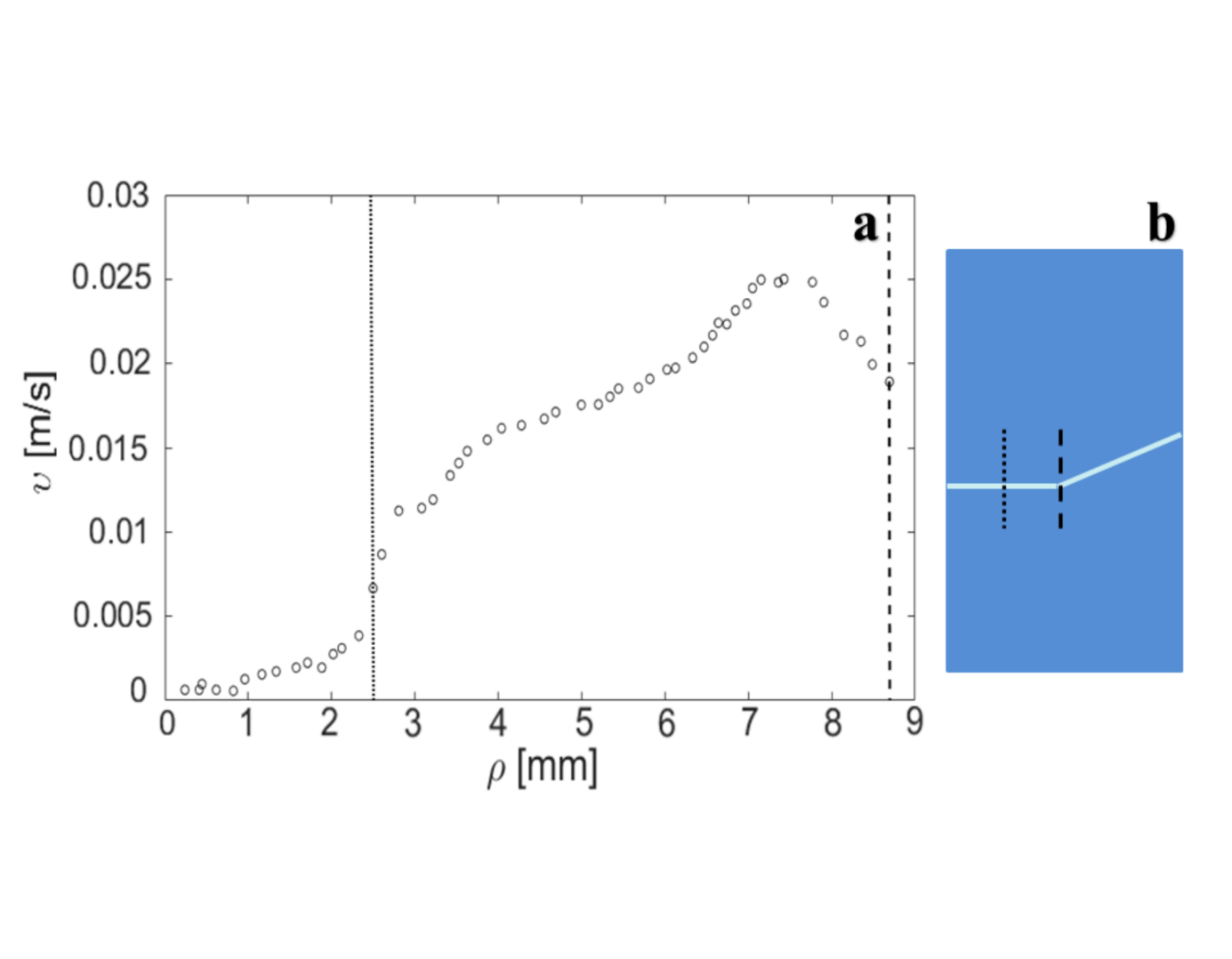


**Supplemental Figure S1.**  **Crack tip velocities.** **a**, Crack velocity, $\upsilon$, plotted against lateral position, ρ, of the crack along the width, *w*, of a dual layer membrane. **b**, Plan view schematic of the dual layer membrane. Lateral positions 25 and 50% across the membrane width are respectively marked in both panels with solid and dashed lines.


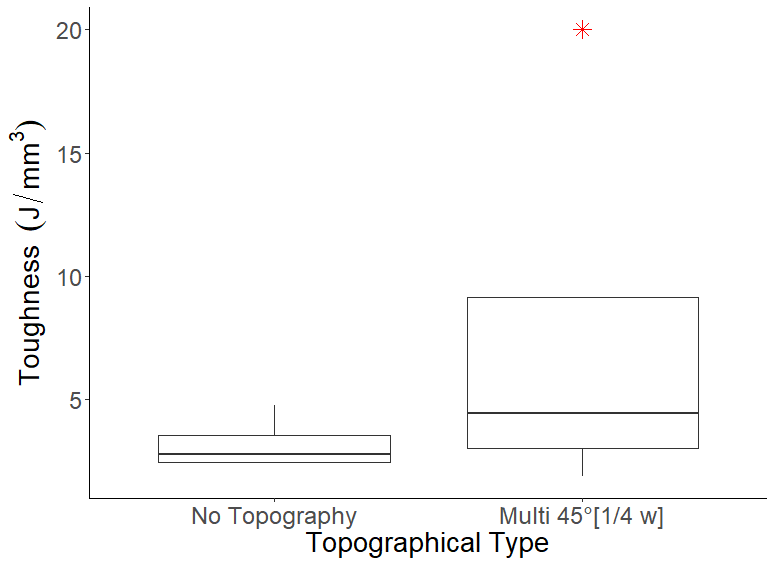


**Supplemental Figure S2.**  **Average toughness for membranes with no embedded topography and membranes with multiple angled sections**. Box plot showing the average toughness for membranes with no embedded topography and membranes with 9 parallel angled sections (denoted here and in Fig. 7 as Multi 45° [1/4w]). Topographical types are distinguished on the horizontal axis by the reorientation angle in degrees, followed by the lateral position across the membrane width, $w$, where the reorientation occurs (in square brackets). Dark horizontal lines within each box denote the median of $n=4$ individual membranes. Whiskers extend to the minimum and maximum of each data set. Box heights denote the inter-quartile range. Red star symbols denote outliers. P-values are determined using a two-tailed unpaired t-test with a t-value of -1.1 and 6 degrees of freedom.

# **
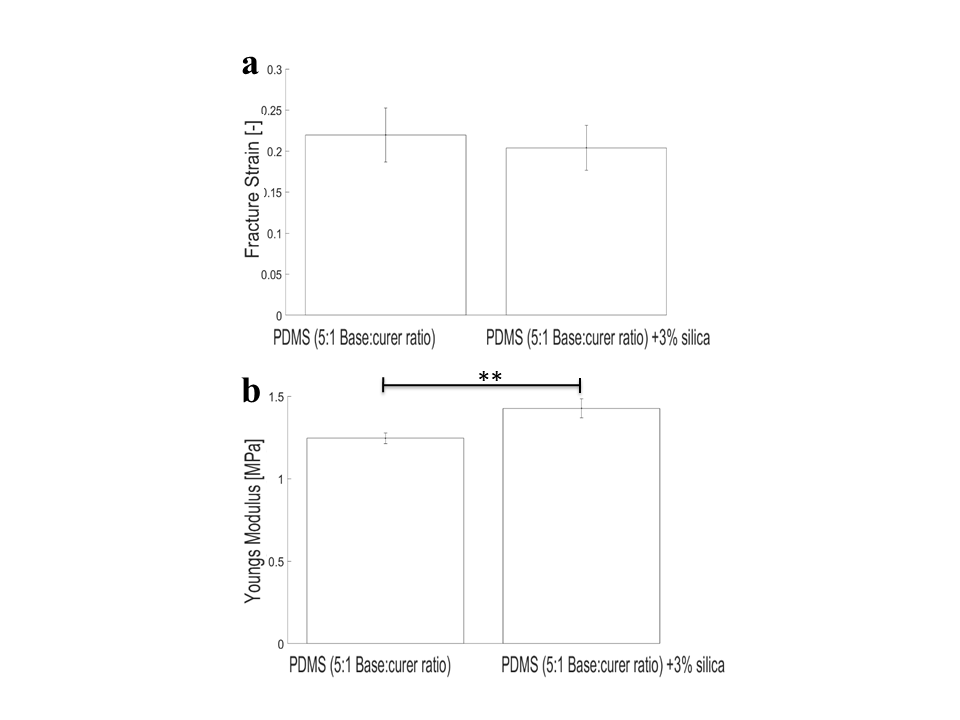
**

**Supplemental Figure S3. Mechanical characterization of PDMS membranes with and without fumed silica inclusions.** **a**, Average fracture strain of homogeneous 19 x 62 x 1.8 mm silicone elastomer membranes made from a 5:1 base to curer ratio of PDMS, and the same ratio PDMS containing 3% by mass fumed silica. **b**, Average Young’s modulus of homogeneous 19 x 62 x 1.5 mm silicone elastomer membranes made from a 5:1 base to curer ratio of PDMS, and the same ratio PDMS containing 3% by mass fumed silica. Error bars denote standard deviations of $n=4$ substrates. Membranes tested here are uniform in thickness and do not contain topographical features. The mechanical properties of the membranes are established using a tensometer (Instron 3342 with 500 N load cell). Samples are strained at a rate of 1mms^-1^ until complete membrane rupture occurs, equivalent to a strain rate of $\dot{\gamma}=0.029$ s^-1^. Statistical significance is established using a two-tailed unpaired t-test for **a, b** with t-values of -0.9 and -4.1 respectively and 6 degrees of freedom.

**Supplemental Movie 1.**  **Multi-channel Fracture.** Video showing a plan view of three 20 mm wide membranes. In the left panel a blank membrane, in the center panel a dual layer elastomer membrane containing eleven embedded parallel topographical channels, and in the right panel a dual layer elastomer membrane containing nine parallel embedded channels. All embedded channels are orientated at an angle of 45° to the parallel section with${l_{1}}/{l_{2}}>0.7$. The samples are strained at $\dot{\gamma}=0.029$ s^-1^ and imaged at 240 Hz. Respectively, the final fractures are displaced approximately 0mm, 7mm, and 12mm from the original longitudinal position.
